# Damage from Carbonated Soft Drinks on Enamel: A Systematic Review

**DOI:** 10.3390/nu15071785

**Published:** 2023-04-06

**Authors:** Angelo Michele Inchingolo, Giuseppina Malcangi, Laura Ferrante, Gaetano Del Vecchio, Fabio Viapiano, Antonio Mancini, Francesco Inchingolo, Alessio Danilo Inchingolo, Daniela Di Venere, Gianna Dipalma, Assunta Patano

**Affiliations:** Department of Interdisciplinary Medicine, University of Bari “Aldo Moro”, 70124 Bari, Italy

**Keywords:** soft drinks, carbonated drink, enamel erosion, tooth, demineralization, dental hypersensitivity, oral pH, tooth decay, bacterial colonization, nutrition

## Abstract

The present study was conducted to analyze the erosive potential of the ever-increasing consumption of carbonated drinks on the dental surface. To identify relevant studies, a comprehensive search was conducted on PubMed, Scopus, and Web of Science covering the last 5 years (2018–2023) using the following Boolean keywords: “soft drinks AND tooth”. Finally, a total of 19 studies were included. The initial search provided a total of 407 items. Nineteen records were finally involved in the inclusion phase, seven of which were in vivo and twelve in vitro. An abuse of carbonated acid substances leads to an increase in the possibility of dental erosion with consequent structural disintegration and reduction of the physical and mechanical properties of the enamel. There is thus greater bacterial adhesion on rougher surfaces, determined by the erosive process, and therefore a greater risk of caries. The pH of most commercialized carbonated drinks is lower than the critical pH for the demineralization of the enamel. Carbonated drinks’ pH and duration of exposure have different deleterious effects on enamel.

## 1. Introduction

Diet and oral health are linked in various ways [1,2,3]. Dental problems are expensive to treat and have a significant negative influence on the quality of life and self-esteem; therefore, much research has attempted to determine the relationship between food, nutrition, and dental disease and to provide dietary suggestions for their prevention [4]. Diet has an impact on tooth formation and later stages [4,5]. Food has the most important nutritional impact on the occurrence of dental caries and enamel degradation [5].

Dental caries is the first cause of disease in developed and developing countries [6,7].

**Figure 1:**
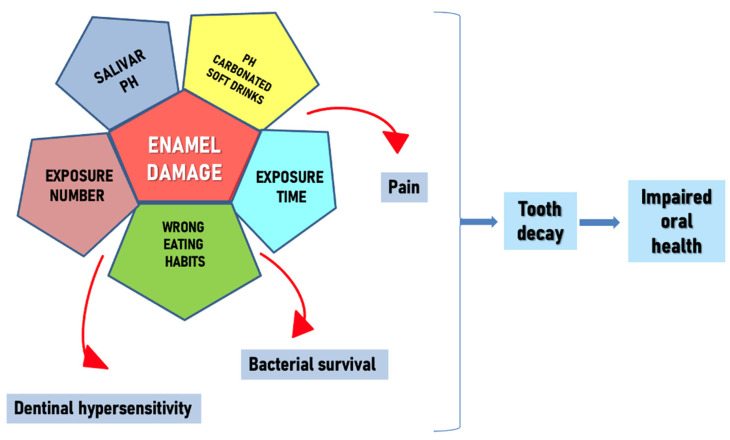
Enamel damage causes.

The etiology is multifactorial: socio-economic, behavioral, genetics, oral pH control, bacterial colonization and adhesion, physicochemical qualities of the tooth, time, carbohydrate intake, and lifestyle [1,8] (Figure 1).

Acids can be of intrinsic and extrinsic origin [9]. These are believed to be among the main causes of dental erosion [9,10]. Acidic foods and drinks, with their low pH, play a key role in the development of erosion [9,10]. However, the pH of a food substance alone is not sufficient to cause erosion [9,11]. Other factors contribute to the erosive process [9]: the presence of calcium, phosphate, and fluorine; behavioral factors such as diet and consumption habits, lifestyle, and excessive acid consumption; and biological factors such as flow rate, buffer capacity, saliva composition, composition and anatomy of teeth, and soft tissue [9,12]. However, the combination of the aforementioned factors with the abrasion process, given in particular by incorrect oral hygiene practices, can be the main cause of the clinical implication of dental erosion [9].

In addition, COVID-19, which saw a rise in unhealthy eating practices and a rise in the use of carbonated beverages, had a significant societal influence [13,14]. On December 12th, 2019, a new coronavirus (SARS-Cov2) emerged in China, sparking a pandemic of acute respiratory syndrome in humans, COVID-19 [15,16,17]. The COVID-19 pandemic represents a massive impact on human health, causing sudden lifestyle changes, through social distancing and isolation at home, with great social and economic consequences [18,19,20]. Optimizing public health during this pandemic requires not only knowledge from the medical and biological sciences, but also of all human sciences related to lifestyle and social and behavioral studies, including dietary habits and lifestyle [15,21]. It has been observed that during the covid period there was a surge in the consumption of carbonated drinks by children, with the consequent increase in the DMFT (teeth decayed missed filled) index [18,22,23].

The intake of carbonated drinks is one of the dangers of dental decay, especially in children and adolescents [24,25,26]. This article analyzes the studies carried out discussing the harmfulness of eating habits, also relating to the habit of carbonated drinks, of the increasingly young population, which gradually tends to worsen [25,27]. The first carbonated drinks were born in the second half of the nineteenth century [28]. Before that, it was customary to drink source water, milk, juices, and beers for refreshment, and only in specific periods of the year, drinks such as syrups, for example, lemon and dandelion made with water and extracts of various fruits [29]. They were, however, seasonal drinks [30]. Changes began in the 1890s when the industries developed beverage-based alternatives to cola extracts; it is thought that this was made by a pharmacist and had medicinal properties [31]. Soon after, much more similar drinks were developed [32]. So-called “soft drinks” are now more readily available because of the industrial manufacture of these beverages and the invention of preservatives [25,33]. Carbonated drinks based on fruit syrups are now very popular for their refreshing taste [25,26,28]. Currently, the increased consumption of fruit juices and carbonated soft drinks, sometimes in place of water, has caused a higher risk of erosion of hard dental tissues [34]. The growing number of manufacturing companies has made these drinks easily available on the market [35]. Lifestyle changes in recent years have continuously increased the demand for these beverages [36,37,38]. This has generated great concern, considering the cariogenic and erosive characteristics of these carbonated beverages [39].

Some studies have considered information on certain types of foods and beverages with a high level of acidity, such as fruit, fruit juices, alcoholic beverages, and carbonated drinks, and the timing of consumption, during main meals or snacks [40].

Enamel erosion, if not controlled, leads to dentinal hypersensitivity and therefore to pain [41,42].

The term dental erosion refers to chemical-mechanical processes in which various extrinsic and intrinsic factors reduce the hard substance of the tooth by removing the softened layer attacked by acids [43,44,45]. Dental erosion can involve both primary and permanent dentition [46,47].

The consumption of soft drinks causes a reduction in the salivary pH [48]. A pH of 5.5 is considered the “critical pH” for enamel dissolution, while it is 6.8 for dentin [17,49,50]. Carbonated drinks have an extrinsic acidity and a pH of up to 2.5 because of the quantity of carbonic acid that is formed with the addition of CO2, which produces the fizz and other acids, such as citric acid, phosphoric acid, and tartaric acid [39,51,52,53,54]. Other important factors, including the type and quantity of acids contained, the buffering capacity, and the temperature of the drink, determine the enamel dissolving capacity of these drinks [55,56,57]. Furthermore, an organic acid produced by the fermentation of sugar in beverages by oral microorganisms present in plaque further leads to demineralization and caries. The characteristics of dental enamel are not common at the macro- and micro-structural morphological level [58,59]. Therefore, the mechanism of enamel erosion must be analyzed from a microscopic point of view [60,61].

The erosive power has been studied from a chemical point of view using the Faurier infrared spectrum (micro FTIR) [62,63].

The aim of this review is to analyze the decent studies in the literature evaluating the possible complications of excessive consumption of carbonated drinks on the dental surface.

## 2. Materials and Methods

### 2.1. Protocol and Registration

A systematic review was conducted according to the Preferred Reporting Items for Systematic Reviews and Meta-Analyses (PRISMA) guidelines for systematic reviews and meta-analyses and the International Prospective Register of Systematic Review Registry guidelines (ID: 405107).

### 2.2. Search Processing

PubMed, Scopus, and Web of Science were searched to find papers that matched our topic dating from 1 January 2018 up to 31 January 2023, with an English-language restriction. The search strategy was built using a combination of words that corresponded to the purpose of our investigation, whose primary objective is the study of the relationship between the use of carbonated drinks and damage to dental enamel; therefore, the following Boolean keywords were used: soft drinks AND tooth (Table 1).

### 2.3. Eligibility Criteria

The following were the inclusion requirements: (1) human in vivo and in vitro study; (2) English language; (3) open access studies; (4) randomized clinical trials; and (5) research on the effects of carbonated drinks on dentin enamel. The exclusion criteria were as follows: (1) animal studies; (2) other languages different from English; (3) not open access studies; and (4) case reports/series, reviews, editorials, and book chapters.

The review was conducted using the PICOS criteria:

- Participants: Teeth of both children and adults were included, in vivo and in vitro.
- Interventions: Considerable consumption of any carbonated soft drink.
- Comparisons: No considerable consumption of any carbonated soft drink.
- Outcomes: Damage to dental enamel.
- Study: Clinical trials on human teeth, both in vivo and in vitro.

### 2.4. Data Processing

Autor disagreements on the choice of articles were discussed and settled. The disagreements were resolved by a third researcher in consensus with the peers.

### 2.5. Risk of Bias Measurement

The risk of bias evaluation was conducted using RevMan 5.5 (Nordic Cochrane Centre, The Cochrane Collaboration, Copenhagen, Denmark, 2014). In accordance with the OHAT Risk of Bias Rating Tool for Human and Animal Studies, the assessment was performed in accordance with the following criteria: randomization sequence, allocation concealment, blinding performance assessment, blinding detection assessment, completeness of procedure description, and selective reporting and other bias. The risks of bias criteria were categorized as adequate, unclear, or inadequate. The selected studies were categorized as low risk of bias with a minimum ratio of 5/7 positive parameters and an absence of a negative outcome. The in vitro studies have been excluded from the analysis.

## 3. Results

**Figure 2:**
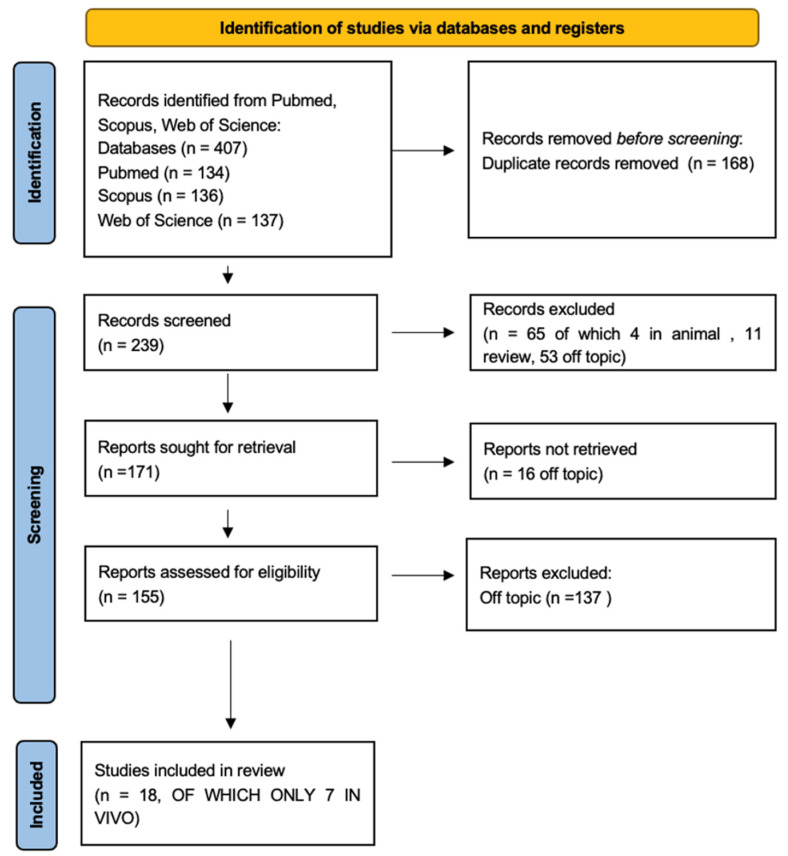
PRISMA flowchart.

The initial search provided a total of 407 items (PubMed n = 134, Scopus n = 136, WOS n = 137), and 239 articles remained after removing 168 duplicates. A total of 156 articles accessed the screening phase, while 83 items were removed because 11 represented reviews, 16 were not free full text, 3 were about animals and 53 were off-topic. From these products, 137 articles were additionally removed for lack of interest in the shown data, and eligibility was assigned to 19 records that were finally involved in the inclusion phase, of which 7 were in vivo and 12 in vitro (Figure 2). The results of each study were reported in Table 2 and Table 3.

### Risk of Bias Measurement

**Figure 3:**
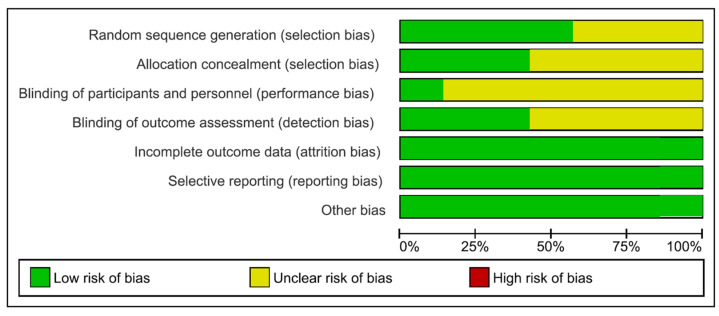

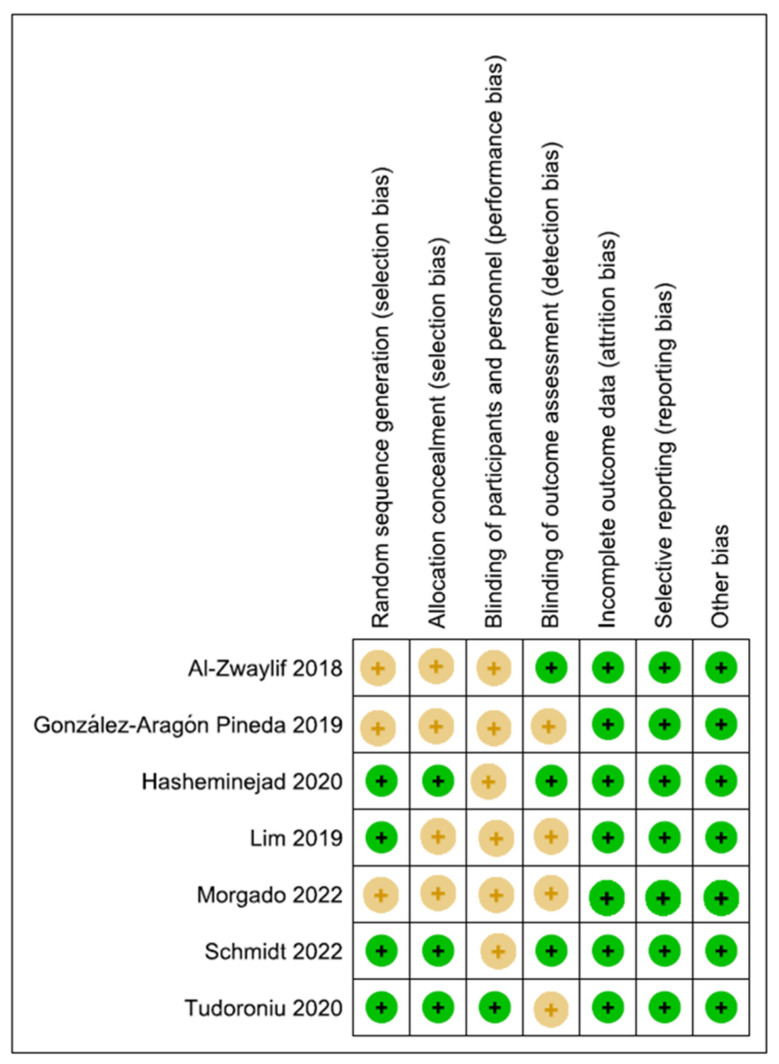
Risk of bias graphs: review authors’ judgements about each risk of bias item presented as percentages across all included studies. In vivo studies: Al-Zwaylif (2018) [40]; González-Aragón Pined et al. (2019) [64]; Hasheminejad et al. (2020) [65]; Lim et al. (2019) [66]; Morgado et al. (2022) [49]; Schmidt et al. (2022) [67]; Tudoroniu et al. (2020) [68].

The risk of bias assessment was conducted for a total of seven in vivo studies and summarized in Figure 3. A total of two papers were considered to have a low risk of bias (Figure 3) by Hasheminejad et al. [65] and Schmidt et al. [67]. The randomization procedure was conducted for almost the ~50% of the papers included. The performance and detection bias were, respectively, the ~15% and the ~40% of the studies included. Due to the wide heterogeneity of the study designs, test groups and population characteristics, a meta-regression was not applicable for further statistical methodologies.

## 4. Discussion

**Figure 4:**
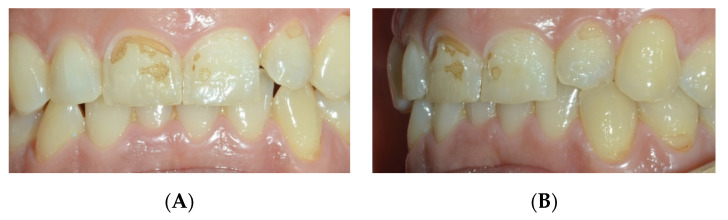

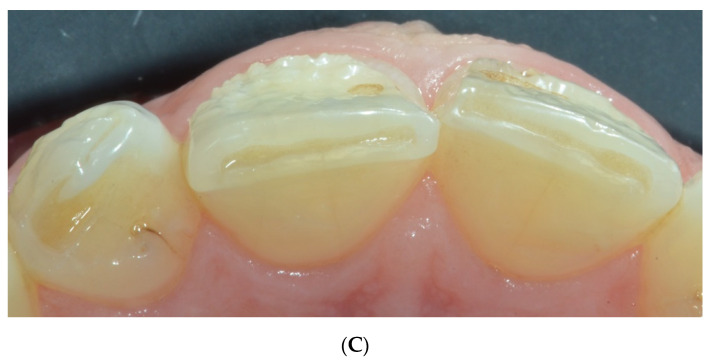
Dental erosion in anterior teeth. (**A**) frontal vision; (**B**) lateral vision; and (**C**) occlusal vision.

Unfortunately, sometimes carbonated drinks, now so common in modern society, replace drinking water [69]. Enamel, while the hardest part of the body, is still a vulnerable structure when exposed to chemicals, such as those found in sodas and beverages [69]. The roughness and hardness of the enamel surface are two reference parameters considered to evaluate the damage of the acids of carbonated drinks, and it has been highlighted that the roughness is the change of the enamel that occurs as an initial erosion phenomenon [69]. Daily consumption of carbonated drinks increases the risk of tooth erosion (Figure 4) [46,76,77].

Erosion is a non-carious lesion of the tooth surface in which there is a continuous loss of enamel and dentin permanently [74]. Although dental erosion is a multifactorial disease and depends on various intrinsic and extrinsic factors, it is strongly influenced by changes in habits and lifestyles [39]. Dental problems are definitely one of the most noticeable health problems in young people [68]. The most frequently consumed acidic beverages were soft drinks and fruit juices [67]. Students aware of dental erosion consumed fewer acidic drinks regularly.

Oral erosion was frequently found on incisors and canines in the upper arch [78]. (Figure 4) Soft drinks acid pH caused changes on the enamel surfaces [75]. The increase in the consumption of carbonated drinks and fruit juices is part of the modern lifestyle all over the world, especially for children and young people [39].

Prevalence data, however, are not homogeneous [79]. Despite this, a trend toward a more pronounced degree of erosion is evident in younger age groups [79,80]. Although dental erosion depends on various factors, intrinsic and extrinsic, it is strongly influenced by changes in habits and lifestyle [30,64].

In some studies, improper nutrition in childhood resulted in a carious tooth surface [66]. These studies demonstrated the use of targeted maximum likelihood estimation in pediatric research because it can address the modeling challenges associated with longitudinal data [66]. Some in vivo studies have evaluated the presence of dental caries in a group of adolescents on the basis of incorrect oral hygiene and food habits [68]. Socioeconomic status and level of education also influence the degree of oral health. Individuals with better education and economic conditions brush their teeth more frequently and have more professional checkups [68]. Socioeconomic factors have also been observed to be considered a risk factor for caries in school-aged children [43,68].

In the studies analyzed, the probability of dental erosion in subjects who had never used sugary soft drinks was 94%, lower than that of daily consumers [65].

Some studies have shown how the erosive effect of carbonated drinks is different between enamel and dentin, considering the histological, organic, and inorganic composition differences of these dental structures [61]. However, the carious process of the enamel must take into account the substantial structural differences of the various portions of the tooth itself which indicate the biodiversity of the enamel [71]. By assessing the fluctuation of the average surface roughness of specific groups of removed teeth following a rigorous immersion protocol in various carbonated beverages selected as a reference, the erosive potential of each drink was determined [69].

**Figure 5:**
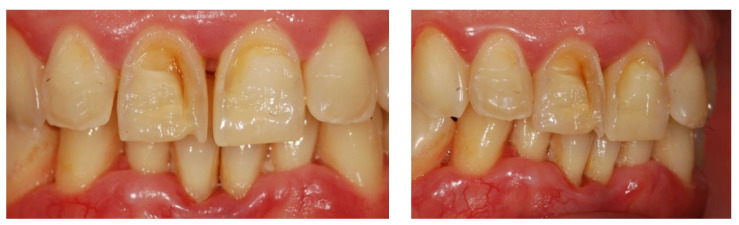
Alteration of the enamel surface.

The structural changes of the enamel, such as hardness and surface roughness, have been studied with various parameters [81]. The acid content of these soft drinks, including citric acid, phosphoric acid, and carbonic acid, lowers the oral pH, making it harmful [39,49,64]. Another important factor increases the probability of demineralization of the enamel, i.e., the prolonged contact time between enamel and drinks [39]. Dental erosion first leads to increased sensitivity and, subsequently, wear of enamel (Figure 5) [59].

Under normal circumstances, saliva tends to increase in the oral cavity in response to the consumption of drinks, and this favors the cleaning of the tooth surface, thus reducing the degree of acidity and, therefore, the risk of erosion [39]. Thus, individuals with a limited salivary flow are at increased risk for enamel erosion [39]. Dental erosion occurs if fluorapatite and enamel hydroxyapatite, two components of the enamel, are exposed to aqueous media with a critical pH below that of fluorapatite, which is pH 4.3–4.5 [70].

Many elements, with various erosive potential, have been examined in the various kinds of carbonated beverages and fruit juices [39]. The erosive potential of a beverage is strongly influenced by its mineral content and its ability to chelate calcium from foods and beverages [70]. Therefore, erosion does not depend only on the pH of the carbonated drink, determined above all by the content of carbonic acid in the form of dissolved carbon dioxide [62]. Dental deterioration has been linked to the daily use of soft drinks. Consumption of soft drinks with meals has been linked to mild or severe tooth decay [40].

The increased amounts of calcium, phosphate, and fluoride in the drinks limited the severity of erosion by changing the solubility of the enamel [82]. The decline in enamel’s surface microhardness and mineral loss were both dramatically halted by the addition of CaGP to the carbonated drinks. Mineral loss reduced as CaGP content in carbonated beverages rose from 2 mM to 10 mM [72]. The studies analyzed performed tests in vitro and then in the laboratory [76]. It was therefore almost impossible to simulate the natural conditions of the mouth [77]. However, to make the in vitro studies more similar to clinical circumstances, some enamel samples were stored for 3 h in saliva prior to testing to allow for pellicle formation [83].

Before swallowing, the maximal quantity of saliva in the oral cavity was 1.19 mL and 0.96 mL for males and females, respectively [53]. Under normal circumstances, human saliva forms a physical barrier, a film, and prevents direct contact between the tooth enamel surface and acidic beverages, thus protecting teeth from erosive attack by acids [45,84,85,86]. However, the erosion tests were carried out without saliva [56]. In addition, they were unable to take into account additional patient-related variables such as salivary flow rate and composition, saliva remineralization capability, and swallowing time [56,87]. The in vitro study and the fixed duration of exposure to the selective test drinks place limitations on the various in vitro studies analyzed [62,88]. The results of the studies provide a relative value of the carbonated beverages’ erosive potentiality [14,62,89]. The concentration of calcium and phosphorus ions in enamel decreased significantly after continuous storage in all beverages tested, but decreased significantly less when saliva was used as a storage medium for the dental specimens [56,90].

Saliva is a fundamental element in physiological conditions due to its buffering capacity and its ability to form a barrier that prevents direct contact between the surface of the enamel and acidic drinks, thus protecting the teeth from acid attacks [39].

Under normal circumstances, salivary secretions increase in the oral cavity in response to drink consumption and remove acid from the tooth surface to limit erosion [56,91].

**Figure 6:**
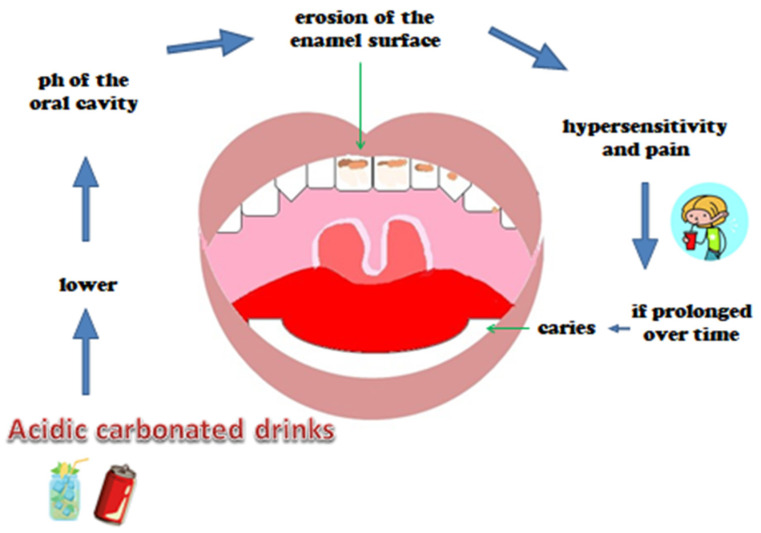
Chain acid-caries.

In order to prevent and regulate erosive wear of teeth (ETW), it is important to understand how the intake of sweet carbonated beverages affects this condition [64]. Most of the drinks available on the market have a higher acidity level than that physiologically tolerated by the tooth, and this involves the superficial demineralization of the enamel, making the tooth more susceptible to fracture and abrasion thus causing hypersensitivity and pain (Figure 6). The roughness of the surface is evaluated on the basis of the porosity and the presence of surface valleys. When the porosity reaches the value of 0.2 μm it is considered harmful, above all causing adhesion of microorganisms [73].

**Figure 7:**
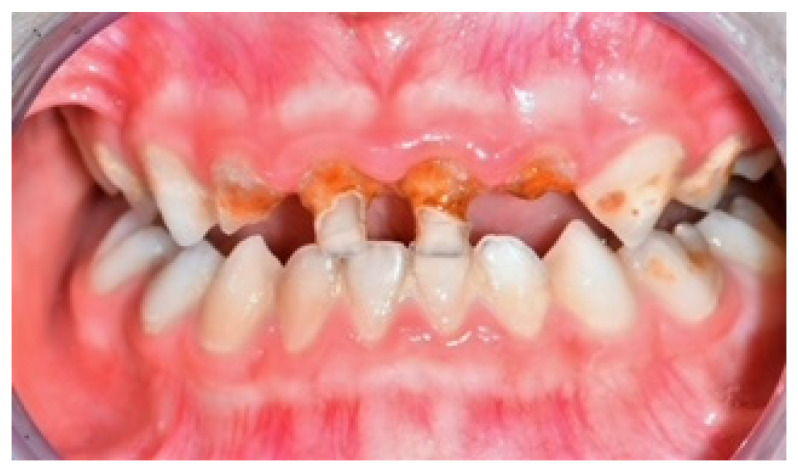
Erosion surface in a child’s teeth.

The increase in the consumption of carbonated drinks in recent times among children and adolescents cause more dental erosion and caries (Figure 7) [24,40,43]. The problem of enamel erosion regards deciduous teeth in an increasingly increasing manner [75]..

In some studies, the variety of reactions of the enamel and dentin of the teeth with a carbonated beverage was examined by studying its crystallographic properties, i.e., after a week of exposure to the alcohol-free drink, samples of cut teeth were examined and the X-ray microdiffraction analysis was carried out using a 100 μm x-ray beam of diameter [61].

Other studies have evaluated the possibility of integrating carbonated drinks with buffering agents such as CaGP, calcium glycerophosphate with properties that can reduce the erosive potential on dental enamel, but the pleasant taste of these drinks, which is the main reason for their wide consumption, outweighs the need to preserve oral health [72].

Soft drink consumption during meals was linked to mild to severe tooth damage [65]. No matter when they were consumed, other acidic meals and beverages were not linked to tooth damage [40]. Certain oral cavity situations, such as the use of acidic drinks, meals, and medicines, endanger not just teeth but also restorative materials in the oral cavity [92,93,94]. In reality, all of the composite resins tested exhibited good resistance to acid assaults, with the exception of glass ionomer cements, which tended to disintegrate [92]. The placement of a protective layer over the latter components may limit the incidence of this occurrence [92].

Regular exercisers are more likely to experience tooth erosion, especially if they consume a lot of sports beverages [95]. In recent years, the ability of biomimetic hydroxyapatite-based mouthwashes and toothpaste to remineralize teeth has been investigated [95,96]. When used alone or in conjunction with a mouthwash that also contains hydroxyapatite, hydroxyapatite toothpaste is an efficient home treatment for tooth erosion in physically active people such as rugby players [95,96]. In several research, the pH of commercial beverages such as sports and energy drinks, water, fruit juices and beverages, soda, milk, tea, and coffee was measured using a pH meter [60,97]. Moreover, gravimetric analysis and the Vickers hardness tester were used to assess the effect of five highly acidic drinks on weight loss and surface hardness of human teeth samples [60,97,98]. Sports and energy drinks, fruit juices, and carbonated soft drinks were in the most acidic beverage categories, considerably reducing enamel surface hardness owing to mineral loss [60,95,98]. Sports participation was found to be substantially connected to erosive injuries [99]. Various physical activities that necessitate certain food diets are the strongest predictors of erosive injuries among teenagers [60,99]. Professionals in water sports, for example, are about 14 times more likely to suffer from erosive injuries [99]. In general, about half of all people who participate in sports have had tooth erosion, and even more than half have consumed sports drinks on a regular basis [100]. Consuming water as your main beverage decreases your chances by roughly 70% [99].

Studies concerning the diffusion of the dental erosive process in the world have shown that milk was one of the least cariogenic drinks. Indeed, over time, many studies have found preventive properties of caries in some dairy products, determined by the presence of fats and proteins [65]. Milk intake reduced dental caries, however, drinking soft drinks increased the amount of dental caries caused by “enamel erosion” [65].

The best prevention that can be implemented in the modern population is to increase awareness that the habitual consumption of carbonated drinks is a determining part of dental erosion [67].

In fact, it was found that the subjects with the best knowledge of the problem of damage to the teeth caused by bad eating habits consumed the least amount of carbonated drinks. It has also been noted that there is generally little information from health professionals who should instead motivate the population to change the behavior of consuming acidic drinks [67]. In this regard, some recommendations at a political level to discourage the consumption of sugary drinks, such as marketing restrictions, pricing strategies, and the reformulation of soft drinks, would be useful. Actions that would lead to improved oral and general health were widespread [67].

This study presented many limitations in the research. The analyses were done almost exclusively in vitro, with extracted dental elements. In fact, the results obtained should be reinterpreted and associated with the in vivo situation in which saliva could reduce the erosive capacity of carbonated beverages due to its buffering power. The in vivo studies analyzed are represented by the evaluation, with questionnaires, of dietary and lifestyle habits in different countries of the world. In addition, the calculation of bias was not applicable; therefore, no assessment of the quality of the studies was made. Another limitation of the research was the analysis in a relatively short time range, with dental erosion from carbonated soft drinks being a rather recent issue.

## 5. Conclusions

The increasing consumption of carbonated drinks has heightened concerns about oral health. Dentists and medical professionals should aggressively educate the public about dental erosion and encourage them to adopt healthier eating habits. Saliva pH is important for the health of tooth enamel. People with little saliva and a habit of frequently consuming acidic beverages are at increased risk for enamel erosion. The basic recommendations are to drink water as the first choice and eat fresh fruits as an integral part of a healthy and balanced diet. Health professionals should motivate the population to change their behavior regarding the consumption of acidic drinks, and recommendations should be made at the policy level to discourage the consumption of sugary drinks. Interventions that would improve oral health and overall health are widely available.

## Figures and Tables

**Table 1:** Database search indicators.

**Articles screening strategy**	KEYWORDS: A: soft drinks; B: tooth
	Boolean Indicators: A AND B
	Timespan: 2018–2023.
	Electronic databes: Pubmed; Scopus; WOS.

**Table 2:** In vivo studies descriptive summary.

Authors (Year)	Type of the Study	Aim of the Study	Materials	Results
Al-Zwaylif et al. (2018) [40]	Study in vivo	Explore the interrelationship between -type and timing of dietary acid intake and -tooth wear.	3586 participants. Data collected: -on four different types of acidic meals, -on the time at which they were consumed, -on the amount of surface area with moderate to severe tooth wear, and -on the type and -timing of acid consumption in the diet.	Dental deterioration has been linked to daily use of soft drinks. Consumption of soft drinks with meals has been linked to mild or severe tooth decay. Other meals and acidic drinks were not linked to tooth deterioration, regardless of when they were consumed.
González-Aragón Pined et al. (2019) [64]	Cross-sectional study in vivo	Evaluate the relationship: use of various drinks–erosive tooth wear.	To calculate ETW (Erosive Teeth Wear), a questionnaire was used to monitor the frequency of beverage intake, which included - water, - milk, - natural fruit juices, - hot drinks, and - soft drinks.	Consuming milk and milk derivatives might serve as a dietary substitute for sugary carbonated beverages in order to help avoid ETW.
Hasheminejad et al. (2020) [65]	Study in vivo	Valuate association beverage intake pattern–dental caries–tooth erosion.	A questionnaire was used to determine normal drinking frequency of 600 adolescents.	Adolescents have a propensity to drink harmful beverages. Milk consumption was shown to be protective against dental caries; soft drinks were linked to increased tooth erosion and dental caries.
Lim et al. (2019) [66]	Study in vivo	The long-term impact of soda intake on dental cavities in young children can alter. Assessing a dynamic impact might be difficult due to follow-up loss and time-varying confounding. The goal of this work is to show how the targeted maximum likelihood estimate technique may be used to overcome obstacles with longitudinal data analysis and estimate the dynamic effect of soda consumption on pediatric caries.	995 pairs of caregivers. The task was to monitor the tooth surface of the children. Variables included -caregiver smoking, -oral health fatalism, and -social support. Children who drank lots of sodas had more cavities than those who did not. The association between soda drinking patterns and caries was investigated using targeted maximum likelihood estimation.	Improper nutrition in childhood resulted in a carious tooth surface. The study demonstrates the use of targeted maximum likelihood estimation in pediatric research because it can address the modeling challenges associated with longitudinal data.
Morgado et al. (2022) [49]	Study in vivo	Prevent the growing issue of dental erosion, especially in the most at-risk patients evaluating the pH values of bottled water and inform patients and clinicians about its erosive potential.	Ph analysis of 105 types of bottled water analyzed: -32 of these were carbonated water; -73 were still water.	The pH of several tested waters is below the safe level for makeup and/or teeth, implying that they are more dangerous to consume than others.
Schmidt et al. (2022) [67]	Study in vivo	Determine the correlations between -sociodemographic characteristics, -awareness and knowledge of dental erosion, and -beverage consumption behaviors by measuring the awareness of dental erosion.	418 students completed an online survey.	The most frequently consumed acidic beverages were soft drinks and fruit juices. Students aware of dental erosion consumed fewer acidic drinks regularly. Most international students knew less about dental deterioration. Older students, who were studying health subjects, accurately identified more types of acidic beverages.
Tudoroniu et al. (2020) [68]	Study in vivo	Determine the presence of caries among adolescents and analyze the relationship between oral hygiene practices and consumption of sugary foods.	650 adolescents answered a questionnaire to analyze the correlation between -the DMFT index, -oral hygiene, and -food habits	Adolescents continue to have a significant prevalence of caries determined by their eating habits.

**Table 3:** In vitro studies descriptive summary.

Authors (Year)	Type of the Study	Aim of the Study	Materials	Results
Al-Amri et al. (2021) [69]	Study in vitro	The enamel exposed to sweet drinks undergoes microscopic changes due to -pH, -time of exhibition, and -other ingredients in the drinks.	three sets of extracted teeth immersed: -in sweet drinks and -in saliva presented, with the use of a profilometer, alterations in tooth surface roughness.	Exposure to sweet drinks increased surface roughness on teeth.
Arafa et al. (2022) [70]	Study in vitro	Dental dentin and enamel respond to carbonated soft drinks.	After one week of exposure to soft drinks, teeth showed, microscopically and with X-ray microdiffraction analysis, a wide range of enamel decay.	Soft drinks caused high erosive effect on the enamel surface of teeth, while milk showed no difference from saliva.
Charpe et al. (2019) [59]	Study in vitro	Assess and contrast the solubility of tooth enamel after exposure to alcoholic beverages across various time periods.	Three distinct beverages were tested for enamel solubility at various time intervals, with extracted teeth. Calcium released into the drinks was analyzed and determined using a semi-automatic analyzer and the Calcium Reagent Set.	Considerable mean calcium is lost beacause of -soft drinks and -beer, whiskey and hard drinks.
Gotouda et al. (2017) [61]	Study in vitro	Reaction of different types of dentin and enamel to carbonated beverages.	X-ray microdiffraction analysis showed a wide range of white stain areas in the enamel, ranging from barely perceptible to nearly complete decay.	This research provides fundamental crystallographic information that will soon be used in preventive dentistry.
Kono et al. (2019) [71]	Study in vitro	Micro-FTIR spectroscopic analysis of teeth sections to clarify chemical processes of dental caries.	X-ray microdiffraction analysis showed a wide range of white stain areas in the enamel, ranging from barely perceptible to nearly complete decay.	This study demonstrated the range and normalcy of dental enamel features.
Manaswini et al. (2020) [72]	Study in vitro	This study’s objective was to compare the mineral loss and surface microhardness of enamel exposed to carbonated drinks with and without calcium glycerophosphate (CaGP).	The surface microhardness of 40 samples of enamel was assessed to determine mineral loss, using -four cycles of intermittent blotting and -spectrophotometric analysis	The decline in the enamel’s surface microhardness and mineral loss were both dramatically halted by the addition of CaGP to the carbonated drinks.
Panic et al. (2019) [62]	Study in vitro	Ascertain how carbonated beverages affected enamel and dentin at various times.	SEM was used to assess and take pictures of 20 samples after: -60 min, -24 h, and -7 days of exposure to the drinks. ANOVA was used to examine the data.	The pH values of the drinks were below the critical pH of the enamel: after as little as 60 min of exposure there were signs of erosion.
Paula et al. (2019) [73]	Study in vitro	Evaluation of: -pH, -acidity, and -erosive potential of juices.	15 third molars were surgically removed and were submerged in a solution of juices and citric acid for four days. Samples were examined for roughness and microhardness before and after the erosive cycles. ANOVA was used to analyze variance.	Juices have an acidic pH, which can lead to erosiveness.
Ramya et al. (2020) [74]	Study in vitro	Examine how soft drinks affect removed teeth’s demineralization.	Teeth that had been extracted. Their weight was determined using an electronic balance. Ten teeth were placed in each serving of soft drink. After a certain amount of time. Their weight was again analyzed The change in weight was noted and the results were interpreted. The investigation revealed that the teeth that had been exposed to fizzy drinks had less weight due to soft drink ingredients eroding their mineral composition.	Carbonated and non-carbonated drinks have different effects on tooth structure.
Shroff et al. (2018) [39]	Study in vitro	erosive potential 20 drinks should be evaluated.	The quantity of sodium hydroxide needed to increase the pH of each experimental beverage to 5.5 or 7 was used to gauge its acidity, and saliva was used to preserve enamel samples. The amount of weight loss on the piece of enamel was determined across time periods.	The experimental beverages had higher acidity values than the packaged fruit juices, resulting in significantly different weight loss after 6 and 24 h of immersion in carbonated beverages.
Sooksompien et al. (2022) [75]	Study in vitro		Children had their 45 first molars removed and submerged in commercial soft drinks or deionized water, resulting in morphological alterations to the enamel surface.	Soft drinks acid pH caused changes on the enamel surfaces.

## Data Availability

Not applicable.

## Abbreviations

DMFT	Decay, Missed and Filled Teeth (index)
ETW	Erosive Teeth Wear
